# The Value of Interventions Aimed at Improving the Patient Experience: Systematic Review of Economic Impacts and Provider Well-Being Outcomes

**DOI:** 10.3390/healthcare13131622

**Published:** 2025-07-07

**Authors:** Tiago S. Jesus, Dongwook Lee, Brocha Z. Stern, Manrui Zhang, Jan Struhar, Allen W. Heinemann, Anne Deutsch, Neil Jordan

**Affiliations:** 1 Division of Occupational Therapy, School of Health and Rehabilitation Sciences, College of Medicine, The Ohio State University Wexner Medical Center, 453 West 10th Ave., Columbus, OH 43210, USA; 2 Center for Education in Health Sciences, Institute for Public Health and Medicine, Feinberg School of Medicine, Northwestern University, Chicago, IL 60611, USA; manruizhang2020@u.northwestern.edu (M.Z.); neil-jordan@northwestern.edu (N.J.); 3 Department of Physical Medicine and Rehabilitation Medicine, Korehab Clinic, Dubai P.O. Box 505169, United Arab Emirates; leedavid@bu.edu; 4 Center for Child Development Center & Research, Sensory EL, Yeosu 59640, Jeollanam-do, Republic of Korea; 5 Department of Population Health Science and Policy, Institute for Healthcare Delivery Science, Icahn School of Medicine at Mount Sinai, New York, NY 10029, USA; brochasternot@gmail.com; 6 Nerve, Muscle + Bone Innovation Center & Oncology Innovation Center, Shirley Ryan AbilityLab, Chicago, IL 60611, USA; jstruhar@sralab.org; 7 Center for Rehabilitation Outcomes Research, Shirley Ryan AbilityLab, Chicago, IL 60611, USA; aheinemann@sralab.org (A.W.H.); adeutsch@sralab.org (A.D.); 8 Department of Physical Medicine and Rehabilitation, Feinberg School of Medicine, Northwestern University, Chicago, IL 60611, USA; 9 RTI International, Chicago, IL 60606, USA; 10 Psychiatry & Behavioral Sciences and Preventive Medicine, Feinberg School of Medicine, Northwestern University, Chicago, IL 60611, USA; 11 Center of Innovation for Complex Chronic Healthcare, Hines VA Hospital, Hines, IL 60141, USA

**Keywords:** patient experience, economics, value, burnout

## Abstract

**Background:** Although improving the patient experience with care is being framed as part of value-based care, the economic and provider well-being impact of interventions for improving the patient experience has not been established. We aimed to synthesize the contemporary (2015–2024) empirical literature on the economic (e.g., costs, revenue) and other value-based impacts (e.g., provider well-being) of patient-experience improvement interventions. **Methods:** Systematic review using six databases of scientific literature (PubMed, EconLit, CINAHL, PsycINFO, DOAJ, and Scopus) supplemented by journal-specific and snowball searches following a registered study protocol (PROSPERO: CRD42022358337). Two independent reviewers performed eligibility decisions and quality appraisals of the study methods and economic assessments, when applicable; the latter was conducted using the Joanna Briggs Institute’s checklist for economic evaluations. **Results:** Out of 1317 unique references, nine were included. Four studies assessed the effectiveness of patient experience improvement interventions (e.g., provider communication training, discharge or transitional support) coupled with economic evaluations; these found statistically significant positive outcomes for both patient experience and economic dimensions—including reduced costs, improved revenue, or additional costs offset by increased revenue. Three additional studies on provider communication training also found statistically significant positive impacts on provider well-being (i.e., reduced burnout) and patient experience improvements. **Conclusion:** These findings shed light on the overall synergistic value of and business case for investments into developing patient experience improvement programs or activities. However, there is room for strengthening this body of knowledge in scope, volume, and method quality, including the need to study the impact on patient experience, provider well-being, health outcomes, and costs (i.e., the *quadruple aim*) in tandem.

## 1. Introduction

Patient experience encompasses a spectrum of valuable patient interactions within the healthcare system, such as easy access to information, timely appointments, and effective communication with healthcare providers [1]. A recent systematic review correlated improved patient experiences with better self-reported outcomes and reduced healthcare utilization [2]. Therefore, monitoring and enhancing patient experience, alongside other quality indicators, is crucial for advancing healthcare quality and value. To facilitate the monitoring of patient experience, the US Agency for Healthcare Research and Quality has established the Consumer Assessment of Healthcare Providers and Systems (CAHPS) program [3]. The CAHPS offers standardized patient experience measures, enabling routine monitoring and performance comparisons across providers. Of note, patient experience measures (or patient-reported experience measures) focus on how the patient experiences the service and care they receive, which conceptually differs from patient-reported outcome measures, which refer to any report that comes directly from the patient about their health condition, without interpretation by a clinician or anyone else [4,5]. Furthermore, patient experience measures conceptually differ from patient satisfaction measures, as the former rely on observable, modifiable aspects of care, while the latter are more subjective and influenced by patient expectations—hence with lower value for both quality assessment and improvement [6]. The routine use of the CAHPS or other patient experience measures can influence consumer choice and encourage quality improvement. For example, the Centers for Medicare & Medicaid Services (CMS) use CAHPS measures for case-mix adjusted public reporting of patient experience ratings, aligning with other quality indicators [7]. Furthermore, the CMS’s Hospital Value-Based Purchasing (VBP) Program, initiated in 2013, incorporates value-based incentives, including CAHPS-measured patient experience performance, to reward providers for key quality indicators [8].

Following value-based incentives and responding to market pressures such as customer loyalty, public reporting of patient experience data, online reviews, and social media accounts [9], healthcare systems and providers are actively striving to enhance their patient experience performance. To achieve this goal, healthcare organizations have various options. These include investments in patient experience improvement programs, engaging in quality improvement activities (e.g., in-service communication training [10]), and implementing new structures, such as patient experience offices [11], to support these improvement programs.

This review is developed from the standpoint that improving patient experience can lead to a return on investment. For example, improvements may lead to enhanced incentive payments under a value-based purchasing program and increased patient volume due to factors such as consumer loyalty, positive word-of-mouth marketing, or favorable online reviews [11,12]. In addition to potential financial returns, initiatives to enhance patient experience can positively impact provider engagement and well-being [13,14].

Viewing healthcare through the lens of relationship-centered care, which recognizes the interdependence of patient–provider interactions [15,16], suggests that organizations fostering initiatives to improve patient experience may also elevate staff engagement, joy at work, and overall clinician well-being. If so, this contributes to the *quadruple aim*, which incorporates patient experience, cost, and provider experience alongside population health outcomes among the goals for quality improvement [17,18]. Furthermore, organizational initiatives emphasizing person-centered tenets can promote staff engagement and activation [19], reinforce a climate of compassionate and relational care, and ultimately improve provider well-being and staff retention [20,21].

However, the value of patient experience improvement activities has not been definitively established or systematically synthesized. While there are systematic reviews that aimed to link patient experiences to clinical safety and improved patient outcomes [2,22], there is a gap in the literature concerning the economic impact of patient experience improvement activities as well as the impact on provider well-being or other value-based outcomes.

We aimed to synthesize the contemporary (2015–2024) empirical literature on the economic (e.g., costs, revenue) and other value-based impacts (e.g., provider well-being) of patient experience improvement interventions. These findings aim to inform healthcare administrators about the potential supplementary value from investments in patient experience improvement activities.

## 2. Materials and Methods

### 2.1. Design

This study refers to a systematic review whose protocol was registered in the PROSPERO database (CRD42022358337). While this systematic review protocol focuses on both the effectiveness and value of health service interventions targeting improvements in patient experience, the systematic review described in this paper focuses on the value-based component. Reporting follows the Preferred Reporting Items for Systematic reviews and Meta-Analyses (PRISMA) guidelines [23].

### 2.2. Eligibility Criteria

Our review addresses two key questions:

(1) What is the economic impact on healthcare organizations of patient experience improvement interventions?
(2) Are there additional value-based impacts, such as improved provider well-being/burnout or reduced healthcare utilization, arising from these interventions?

To answer both review questions, we included empirical studies (e.g., controlled experiments, longitudinal observational studies on the impact of a program/intervention, pre–post test designs) of all activities (e.g., service delivery and other in-service improvement interventions) targeting improvements in patient experience that also reported financial components or other value-based outcomes (e.g., burnout, readmission rates, healthcare utilization). For patient experience data (i.e., study outcomes), we included studies that used standardized patient experience assessments, including validated surveys or surveys that were externally collected and routinely used across providers, including for value-based reimbursement. We excluded studies that did not (1) use standardized measures or inferential statistics for evaluating the patient experience outcomes or (2) report any additional value-based outcomes (e.g., burnout-related healthcare utilization) or financial/economic outcomes (e.g., return on investment). For participants, we included health systems, organizations, providers, networks, settings, or service units, including any health professionals or staff. We excluded health service interventions exclusively delivered by students or clinicians-in-training. Those providing patient experience feedback could be the patient, family/informal caregivers, or any proxy respondents on the patient’s behalf. There were no limits placed on healthcare settings, geography, or health conditions. In addition, we had no requirement for a comparator (i.e., we included studies with inferential pre–post analysis). As planned in the protocol, we excluded studies not reported in English and those published before 2015, as we looked for contemporary evidence accessible to an international readership. Altogether, we covered the time period from 2015 through to 2024.

### 2.3. Search Process

Six scientific literature databases (PubMed/Medline, EconLit, CINAHL, PsycINFO, DOAJ, and Scopus) were searched using a combination of free-text words with indexed terms. We used terms for patient experience or terms related to methods of collecting or improving those experiences (e.g., experience-based codesign, experience rounding). These keywords were combined with a broad set of keywords on health service management and improvement approaches. No restriction was applied to country or health service type. The most recent search was updated until December 2024. Supplementary Table S1 provides details about the complete search.

The databases searches were complemented by targeted searches within specific peer-reviewed journals with a track record of publishing on patient experience activities. These journals include *Patient Experience Journal*, *Journal of Patient Experience*, *Medical Care*, and *Health Expectations*. *NEJM Catalyst Innovations in Care Delivery* was also searched for publications since January 2020, when the journal became peer-reviewed. Finally, we applied snowball strategies (e.g., author tracking, citation tracking) using the final included articles and any related systematic or scoping reviews, including those identified through the initial searches.

### 2.4. Selection of Papers and Data Extraction

Two independent reviewers were used for title-and-abstract screening and full-text selection; two rounds of discussion among the reviewers were sufficient to achieve agreement. The research team constructed a tailored data extraction table, which included data on (a) study characteristics (e.g., study design, objective, country, settings, measures used, analytic approaches), (b) interventions used and their impact on patient experience scores, (c) any economic analyses and their findings (i.e., on financial variables or indicators assessed, sources of these data, economic analysis type and methods used, and the results of these analyses), and (d) any additional analyses and findings on provider well-being/burnout or quality-of-care outcomes. Data extraction was performed by the lead author and then fully verified by another author for accuracy and completeness.

### 2.5. Quality Assessment (Risk-of-Bias Assessment)

We used a two-level quality assessment. First, we assessed the methodological quality of the overall study design according to design type. For controlled experiments, we used the Cochrane-suggested risk-of-bias criteria for Effective Practice and Organization of Care reviews. In turn, for pre–post and observational studies, we used quality assessment tools from the National Institutes of Health. Finally, for the studies that also contained economic assessments, we additionally used the Joanna Briggs Institute’s critical appraisal checklist for economic evaluations. Two independent reviewers performed each assessment, with a third reviewer involved in resolving any conflicts.

### 2.6. Data Synthesis

The study methods, indicators, and quantitative findings, with a focus on the economic and other value-based assessments, were then tabulated and narratively synthesized to address each review question. Due to the heterogeneity of contexts and study designs, meta-analysis was not possible.

## 3. Results

**Figure 1:**
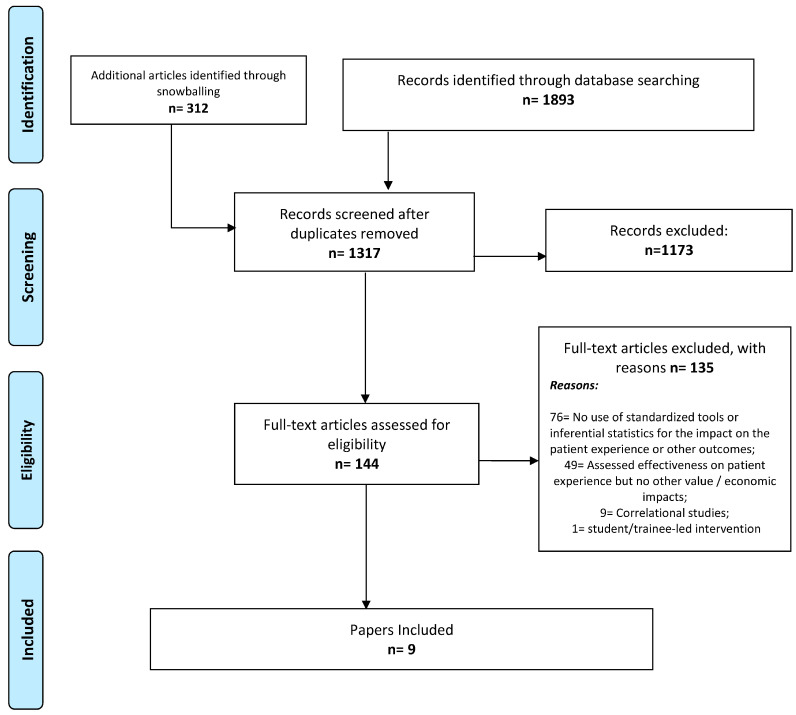
PRISMA flowchart of the review.

Figure 1 provides the PRISMA flowchart of this review. Out of the 1317 unique references, 144 full texts were assessed, and nine were included—all from the USA. Of the studies on patient experience that also assessed other value-based impacts, while some could focus on more than one impact type, five focused on economic impact, three on provider well-being/burnout, and four on healthcare utilization. Two studies that assessed readmission rates also assessed economic impacts, i.e., they fit in both categories.

The methodological quality of the economic impact evaluations ranged from a few methodological concerns, such as the use of estimated versus actual reimbursement [24], to a paper with no details about how the financial results were obtained [25]. Supplementary Tables S2 and S3 provide a synthesis of the methodological quality assessment of each study (for both the underlying study design and the economic evaluation component, where applicable) based on the respective quality assessment checklists. The key methodological features of each paper provide the context for the synthesized findings below for each review question.

### 3.1. Economic Impact of the Patient Experience Improvement Interventions

Table 1 provides synthesized details of the four studies on the improvement of patient experience that also assessed the economic impact—on costs, revenue, operating margin, or combinations of these. All these studies showed significant changes in at least some of their dimensions (e.g., in five of the 11 assessed items [24]).

Below, we synthesize the economic findings and methods, organized by intervention type.

#### 3.1.1. Discharge Support

Two studies focused on this intervention type (Table 1). A controlled before-and-after study with historical controls and adjustment for price changes found that a telephone-based transitional support cost an additional $430 per patient compared to usual care. The increased cost was statistically significant in both the univariate and multivariate models (*p* < 0.01 and *p* = 0.03, respectively). However, the same models showed no significant changes for hospital margin (*p* = 0.25 and *p* = 0.23, respectively) [24], suggesting that improved revenues were offsetting the increased cost of the intervention.

The study found that communication training for the surgeons (*n* = 56) of a children’s hospital increased hospital revenue (payments by 25% and charges by 21%) after adjusting for yearly price changes. In the same study, a small, controlled subgroup analysis (surgeons; *n* = 8) showed that payments and charges increased further (by 71% and 113%, respectively) for the plastic surgeons who received communication training, while revenue decreased for the surgeons who did not receive the training [10].

#### 3.1.2. Patient Experience Office

Finally, we included a secondary analysis (i.e., an observational, retrospective, comparative study) of the financial impact of adding and operating a patient experience office within a healthcare organization’s structure, applied to a national sample of 132 hospitals [11]. Of note, a patient experience office is a formal, cross-departmental organizational unit dedicated to assessing, analyzing, and optimizing the entire patient journey and patient experience performance of a healthcare organization. The study found a 1.4% reduction in operating costs for each added year of the office’s operation. The article also reported that supplementary key informant interviews revealed that the associated positive financial results are likely a result of efficiency in communication training, improved outcomes, greater patient volumes, and better value-based reimbursements [11]. Altogether, these results suggest a potential positive financial impact for organizations that run patient experience offices and continue doing so over time.

### 3.2. Other Value-Based Impacts of Patient Experience Improvement Interventions

Seven studies on the improvement of patient experience with care also assessed other value-based impacts, such as provider well-being/burnout (*n* = 3) and readmission rates (*n* = 4). Of note, two of the latter [24,25] also assessed economic impacts and, therefore, have some components described in Table 1.

#### 3.2.1. Provider Well-Being/Burnout Outcomes

Table 2 summarizes the three studies assessing the impact of patient experience improvement interventions on provider well-being or burnout, all using a pre–post design and employing physician communication training interventions. Two pre–post studies were relatively large (physicians trained = 104 [26] and 947 [14]), and the smaller study (physicians trained = 30) was part of a randomized controlled trial (RCT)—i.e., a pre–post study for the burnout outcomes and RCT for the patient experience outcomes [13].

All three studies found positive impacts on the patient experience of care, although the smaller study found them only for patient experience scores (i.e., not on percentile ranks, among peer providers) [13]. All three also found improvements in provider well-being or burnout. The smaller study found significant improvements in two of the subscales of the Maslach Burnout Inventory [13], while the largest found gains for all three subscales, with improvements on two subscales sustained after three months [14]. Finally, the remaining study found statistically significant positive impacts on three out of five burnout subscales measured with the Professional Fulfillment Index (Table 3), although not if Bonferroni corrections were applied (supplementary Table S2) [26].

#### 3.2.2. Healthcare Utilization (e.g., Readmission Rates, Emergency Room Visits)

Table 3 describes four studies on improving the patient experience with post-discharge support or transitional care that also assessed the intervention’s impact on readmission rates.

One study found statistically significant impacts on healthcare utilization in the planned analyses. In a pragmatic randomized controlled trial of transitional care, no significant between-group differences in healthcare utilization (outpatient visits, hospitalizations, emergency room visits) were found within 60 days [27]. In a controlled before-and-after study of telephone-based post-discharge support, no significant between-group difference was found for 90-day readmissions (*p* = 0.21) [24]. In the pre–post study of discharge support, no significant differences in readmission rates were found [25]. Finally, a case–control study found no effects on readmission outcomes from a pharmacist-led, pre- and post-discharge education and medication reconciliation intervention; only in an unplanned subgroup analysis (for the post-discharge component) did the study find pre–post reductions on 30-day readmission rates: 17.3–12.4% (*p* = 0.007) [28].

## 4. Discussion

This review synthesizes the recent peer-reviewed literature spanning 2015–2024, focusing on the synergistic value-based assessments of patient experience improvement interventions. Emphasis was placed on economic impacts, provider well-being, and healthcare utilization outcomes. The synthesized interventions focused on provider communication training or post-discharge and transitional support. These interventions showed effectiveness in improving selected dimensions of patient experience and were coupled with either fiscal neutrality (e.g., costs offset by improved revenue [24]) or positive financial returns, such as improved revenue [10]. Reduced healthcare utilization was not found in the target outcomes of studies on discharge and transitional care processes [24,25,27,28]. Nonetheless, these processes resulted in economic benefits [25] or neutrality [24]. These results align with secondary correlational studies, demonstrating positive associations of patient experience scores with health organizations’ financial performance [25,29,30].

From a broader value-based perspective, interventions targeting provider communication have been shown to yield gains in provider well-being and reduce burnout [13,14,26]. Through the lens of relationship-centered care, it is hypothesized that improving patient–provider communication can positively impact patient as well as provider experiences, which are viewed as dynamically interconnected [15]. The improvement mechanism may involve fostering staff engagement with compassionate and relational care, contrasting with instrumental organizational climates that can negatively affect provider well-being and sense of accomplishment [19,20,21,31,32].

Initiatives for improving patient–provider communication may have a high synergistic potential and even potentially contribute to all four components of a value-based perspective focused on the *quadruple aim*: population health, cost, patient experience, and provider well-being [17,18]. Here, we found that these initiatives are associated with positive financial results, patient experience, and provider well-being, and synergistically so. In turn, in the broader literature, better patient experience scores have been correlated with improved self-reported patient health outcomes [2], including through enhanced shared decision-making [33]. Therefore, an enhanced value proposition may apply to organizational activities that aim to improve patient–provider communication.

Additionally, enhancing provider well-being can offer significant value to healthcare organizations grappling with challenges in recruiting, retaining, and maintaining an engaged and productive staff. High vacancy and turnover rates entail various costs, including recruitment and retention expenses, continual retraining of new staff, the inability to meet heightened demands due to staff shortages, and potential risks to reliability resulting from uncoordinated team processes [17,34]. Notably, the potential benefits of these factors were not assessed or quantified in the reviewed literature and may be further avenues for research.

Similarly, the literature did not explore the potential reduction in litigation costs despite the plausibility of such benefits. Improved communication practices, a frequent determinant of litigation, could lead to diminished legal costs or malpractice premiums [35]. Lastly, there were no included studies on patient experience improvement activities beyond provider communication or post-discharge support (e.g., real-time patient experience feedback [36,37]). Exploring these avenues could provide further opportunities to investigate the synergistic value of activities aimed at enhancing patient experience.

While the literature primarily focuses on the United States, this trend may stem from a combination of factors. The CMS’s Value-Based Purchasing program offers incentives to U.S. health providers based on quality and patient experience metrics, potentially motivating health organizations in the U.S. to invest in and study patient experience improvement activities. However, caution must be taken regarding the generalizability of the results from this review to other contexts, where the same incentives and returns may not apply. Additionally, the U.S. operates under a consumer-based healthcare delivery system, where consumers’ choices for providers are influenced by lived patient experiences, word-of-mouth recommendations, online reviews, and comparison websites [9]. This dynamic places the financial strength of healthcare organizations at risk based on their performance in patient experience. Consequently, these incentives are likely drivers for increasing organizational investments in patient experience improvement structures and activities in the U.S. [11], fostering an enhanced interest in studying the business case for such investments.

## 5. Limitations

These results should be taken with caution, apart from the generalizability outside of the U.S. context. The volume of the literature was not large, and controlled study designs were rare. In addition, we did not conduct a meta-analysis due to the heterogeneity of interventions, contexts, and study designs. We only covered English-language peer-reviewed literature, while many economic analyses of patient experience improvement activities may be in the grey literature or even in unpublished, internal analyses. Many health sector contexts, such as primary care settings and safety-net hospitals, were not adequately represented in the reviewed literature. Furthermore, the economic analyses exhibited a spectrum of methodological rigor, ranging from no substantive risks of bias to a complete underreporting of methods, resulting in significant variability. Collectively, these methodological weaknesses and variations in study designs pose challenges to the robustness of the body of knowledge derived from this review.

## 6. Conclusions

The findings of this review, based on the literature from the U.S. context, underscore the potential financial and employee well-being benefits associated with investments in activities targeting and effectively achieving improvements in patient experiences with care. These results contribute to the value-based argument for healthcare administrators to prioritize investments in patient experience improvement activities, with a particular emphasis on enhancing patient–provider communication.

However, it is essential to approach these findings with caution, considering the substantive potential for strengthening this body of knowledge in terms of scope, volume, and methodological quality. Opportunities exist for incorporating additional economic variables, such as malpractice litigation costs and factors secondary to employee engagement and retention. Additionally, further research could explore the impact on the quadruple aim, encompassing patient experience, provider well-being, clinical effectiveness, and costs, particularly when examined in tandem.

## Figures and Tables

**Table 1:** The costs and benefits of interventions aimed at improving the patient experience of care, ordered by date of publication.

		Patient Experience Component		Economic Component		
	Design and Context	Intervention (Synthesis)	Patient Experience Outcomes (Synthesis)	Economic Indicator(s) and Data	Economic Analysis and Methods	Economic Outcomes
(Thum, Ackermann et al., 2022) [25]	Pre–post test, 12 months each. Academic hospital and its community hospital affiliate.	*Discharge support*: nurses were trained in *teach-back*; workflow was redesigned. A new discharge summary was created, linked to a *hard stop* in the electronic health record.	Improved top box %: care transitions, 52.4–54.5% (*p* < 0.001); discharge information, 87.4–90.1% (*p* < 0.001). Improved percentile rank: 45.2–74.3 (*p* = 0.020) for discharge information.	*Revenue*; unspecified internal organizational data	*Hospital revenue* impact analysis, pre- and post-intervention, unspecified methods.	The intervention had a positive impact on the value-based purchasing program, with an estimated savings of $75,000 compared to the pre-intervention expectations due to better patient experience scores in the care transitions and discharge information domains.
(Schreiter, Fisher et al., 2021) [24]	Controlled before and after, historical cohort of controls. Academic hospital.	*Post-discharge support:* Telephone follow-up delivered by nurses after hospital discharge for patient education, medication reconciliation, or remediation care (e.g., same-day clinic appointment), if required.	Intervention got greater % of top-box scores in 5 of the 11 items: asking about having the needed help (100% vs. 93%, *p* < 0.01), educational materials (68% vs. 55% *p* < 0.01), understanding of responsibilities (69% vs. 59%, *p* = 0.02), instructions on who to call with post-discharge questions (76% vs. 69%; *p* = 0.04), and global experience (57% vs. 46%, *p* = 0.02).	*Costs*: total hospital costs and transitional care expenses (new nurses’ salaries and fringe; infrastructure investments negligible). *Margin:* estimated payer reimbursements minus the total hospital costs.	*Hospital costs and margin impact.* Fiscal data were converted to 2017 US dollars using the Consumer Price Index. Wilcoxon rank-sum test was used to compare the groups’ fiscal data. Predictive multivariable models of the cost and margin for index admission, 90-day readmission, and aggregate. Covariates were those with statistical and clinical relevance (e.g., age).	The intervention cost +$430 per patient; the rank-sum comparison for the aggregate 90-day admissions showed between-group differences for the hospital cost ($25,827 vs. $22,814, *p* < 0.01) but not the margin: $4.698 (95% CI: −2.85–21,100) vs. $7.027 (−1.36–20,734), *p* = 0.25. The multivariable model showed similarly significant results (cost differences: *p* = 0.03; margin differences: *p* = 0.23).
(Abu-Ghname, Davis et al., 2021) [10]	Pre–post test, retrospective, one site; controlled subgroup analysis for plastic surgeons. Children’s hospital.	*Communication training*: 5.5 h course led by two volunteer practicing clinicians trained in-house (in facilitation in a model of relationship-centered care). The course featured role-playing of the communication skills.	Improvements in the scope of provider recommendation (90.7–94.1; *p* < 0.001), language (90.9–94.0; *p* = 0.007), concern (91.4–94.1; *p* = 0.007), decision-sharing (91.8–94.3; *p* = 0.001), and information (94.0–95.4; *p* = 0.031).	*Revenue*, in *charges* (the amount billed by the hospital) and in *payments* (the amount of reimbursements received). Internal organizational data adjusted for price increases.	*Hospital revenue*, pre–post impact: total charges and overall payments (plus the number of distinct patients and encounters). To control for price changes, revenue was compared after controlling for the calendar year 2016 per-unit charges (Current Procedural Terminology).	Payments increased by 25%, and charges increased by 21%—clinical encounters increased by 26%, and the number of patients increased by 26%. Specifically for the subgroup of plastic surgeons, the participants reported a 113% increase in charges and a 71% increase in payments, whereas the controls had decreases of 10% in charges and of 4% in payments.
(Sharma, Chandrasekaran et al., 2020) [11]	Retrospective comparative study. National sample of hospitals. Key informant interviews (1 hospital).	*Office of patient experience* (OPX): patient experience office as a new administrative structure with its own budget and staff and a head who is an executive board member (versus a hospital without that structure).	A 1.95% increase was found in experiential quality per year of operation (*p* < 0.05), more so for hospitals with high vs. low patient complexity (6.5% vs. −0.3%, *p* < 0.05).	*Operating costs*. Any expenses incurred in every aspect of a hospital’s operations, including salaries, supplies, and administrative expenses. CMS’s Cost Reports	*Hospital operating cost* impact: proxy for the cost of setting up and running an OPX. Once computed from CMS’s Cost Reports, operating costs were divided by the number of beds to normalize it for size. A natural log of cost per bed was used to reduce the impact of outliers. Fixed-effects instrumental variable regression with years of OPX operation as the predictor.	Years of operation were weakly associated with reduced operating costs (*b* = −0.18, *p* < 0.10). This translates into a 1.4% operating cost reduction per added year of operation. Interviews suggest efficiency in training, improved outcomes due to better provider communication, greater patient volumes due to satisfaction and word of mouth, and better value-based reimbursement.

**Table 2:** Well-being outcomes from interventions aimed at improving the patient experience of care, ordered by date of publication.

		Patient Experience Component		Well-Being Outcomes	
	Study Design and Context	Intervention (Synthesis)	Patient Experience Outcomes (Synthesis)	Outcome(s) Type and Measure	Outcomes
(Altamirano, Kline et al., 2022) [26]	Pre–post test, 3 months post-intervention, four sites. Academic hospital—four sites, multi-departmental.	*Communication training:* training (8 h) in workshops (*n* = 48; 14 seats each), led by trained peer physicians. After nomination by department chairs, a board selected instructors based on 6 criteria, e.g., patient experience scores, thought leader for communication. The instructors had training toward certification. The trainees applied the skills to cases elicited during the workshop, followed by small group feedback. Continuing education credits were provided.	Top-box scores increased from 82.8% to 84.5% (*p* < 0.0001). The odds of receiving a top-box score 6 months after the program vs. before it (1.11, *p* = 0.01) and >6 months (1.15, *p* < 0.0001) also increased. Gains persisted in a propensity score-weighted analysis (1.09, *p* = 0.04; 1.14, *p* < 0.0001). When stratified by site, two of the four had significant improvements.	*Burnout/well-being:* Professional Fulfillment Index subscales: burnout, compassionate self-improvement, professional fulfillment, emotional exhaustion, and interpersonal disengagement	*Burnout* decreased significantly from 35% to 26% (*p* < 0.039). In addition, *compassionate self-improvement* and *professional fulfillment* increased from 37% to 50% (*p* < 0.020) and from 41% to 51% (*p* < 0.034). Scores for *emotional exhaustion* and *interpersonal disengagement* decreased, but the changes were not statistically significant.
(Congiusta, Ascher et al., 2020) [13]	Pre–post test (burnout outcomes) within an RCT (patient experience outcomes), medical practices.	*Communication training:* online provider training weekly for 24 weeks and biweekly conference calls led by top-performing physicians trained in the model and facilitation. The trainees needed to “learn,” “do,” and “share” the successes in conference calls or a web tool. Team-based breakfast for the best performers and a graduation celebration.	The intervention group had a greater improvement in scores compared to controls (median [Q1, Q3] = 1.6 [0.4, 2.4] vs. 0.6 [−1.3, 1.9], *p* < 0.039), but no significant difference in the percentile ranks (median [Q1, Q3] = 4.0 [−27.0, 13.0] vs. −13.0 [−36.0, 12.0], *p* < 0.346).	*Burnout*: Maslach Burnout Inventory and its three subscales: emotional exhaustion, burnout, depersonalization, and personal achievement	Two of the subscales had significant changes. The *depersonalization* score was significantly lower than the baseline—mean difference (SD) of −2.43 (5.30) (*p* < 0.023), and the *personal achievement* score increased (mean difference of 3.10 (3.62); *p* = 0.0007). The decrease in the total burnout score nearly reached statistical significance (*p* = 0.0504).
(Boissy, Windover et al., 2016) [14]	Pre-, post-, and 3 months post-study within a controlled, before- and-after study; hospital and clinician groups.	*Communication training:* 8 h provider training. Each course (<12 physicians each) was facilitated by two clinicians trained in the communication model, adult learning, performance assessment, and group facilitation. Didactic presentations, live or video-based skill demonstrations, and small-group skills practice sessions were followed by skills practice on communication challenge from trainees’ practices.	Clinicians Group: Adjusted *communication* scores were greater for the intervention group vs. controls (92.09 vs. 91.09, *p* < 0.03). Hospital: Adjusted *respect* scores were greater in the intervention vs. controls (91.08 vs. 88.79, *p* = 0.02), but differences were non-significant for the adjusted *communication* scores (83.95 vs. 82.73, *p* = 0.2).	*Burnout:* Maslach Burnout Inventory and its three subscales: emotional exhaustion, burnout, depersonalization, and personal achievement	Following the course, lower burnout was significantly found on all three subscales (*emotional exhaustion*: *p* < 0.001; *depersonalization*: *p* = 0.003, and *personal achievement*: *p* = 0.04). Improvements in all measures except emotional exhaustion were sustained at 3 months.

**Table 3:** Healthcare utilization outcomes from interventions aimed at improving the patient experience of care, ordered by date of publication.

		Patient Experience Component		Healthcare Utilization Outcomes	
	Study Design and Context	Intervention (Synthesis)	Patient Experience Outcomes (Synthesis)	Outcome(s) Type and Measure	Outcomes
(LaBedz, Prieto-Centurion et al., 2022) [27]	Pragmatic RCT, patient-level randomization. Multivariable linear regression models, with a Bonferroni correction for the co-primary outcomes.	*Transitional care:* the intervention group received an intervention during the index hospitalization and for 60 days post-discharge, which included (1) in-hospital visits by a community health worker to assess barriers to health/healthcare and to develop a personalized discharge patient education tool (DPET); (2) a post-discharge home visit by a community health worker to review the DPET; and (3) telephone-based peer coaching.	No significant between-group differences at 30 days in informational support (adjusted difference: −0.01, 97.5% CI: −2.0 to 1.9, *p* = 0.99), or any secondary outcomes such as emotional support [−0.12, 95% CI: −1.5, 1.2, *p* = 0.86] or instrumental support [−0.43, 95% CI: −1.7, 0.93, *p* = 0.53]. An exploratory subgroup analysis showed greater improvements in 30-day informational support for the navigator group participants without health insurance (+11.9, 95% CI: 2.3 to 21.4).	*Utilization*: 14-day outpatient visit, 30-day and 60-day hospitalization or emergency room visits	No significant between-group differences in healthcare utilization (outpatient visits, hospitalizations, emergency room visits).
(March, Peters et al., 2022) [28]	Observational, case–control comparison with retrospective review, single-center, pilot program, hospital-based pharmacy.	*Patient education and discharge support:* pharmacist-led medication reconciliation and education, sensitive to health literacy levels, pre- and post-discharge, following alerts from the electronic medical record system.	Significant improvement in the top-box scores (52.6% vs. 67.3%; *p* ≤ 0.001) in the composite of medication-related CAHPS results and its specific items: “tell you what the medicine was for” (67.7% vs. 81.9%; *p* = 0.018), “describe possible medicine side effects” (37.7% vs. 58.9%; *p* = 0.004), and “understood the purpose of taking medications” (52.3% vs. 63.7%; *p* = 0.035).	*Readmissions:* 30-day readmissions	Non-significant difference in the 30-day readmissions for the complete intervention vs. non-intervention (16.4% vs. 13.3%; *p* = 0.133); an unplanned subgroup analysis for the discharge phone calls (with or without discharge education) showed a significant reduction in the 30-day readmission rates: 17.3% vs. 12.4% (*p* = 0.007).
(Thum, Ackermann et al., 2022 [25]), described in Table 1				*Readmissions:* 30-day readmissions	No significant difference pre- to post-intervention (*p* = 0.69).
(Schreiter, Fisher et al., 2021 [24]), described in Table 1				*Readmissions:* 90-day readmissions	No significant difference between the groups (*p* = 0.21)

## Data Availability

No new data were created or analyzed in this study.

## Supplementary Materials

The following supporting information can be downloaded at: https://www.mdpi.com/article/10.3390/healthcare13131622/s1, Table S1: Full search strategy per scientific database. Table S2: Study design and methodological weaknesses based on the respective quality assessment checklists for the studies responding to the first study question. Table S3: Study design and respective methodological weaknesses based on quality assessment checklists for the studies responding to the second review question.
