# Supplementary material for: The Value of Interventions Aimed at Improving the Patient Experience: Systematic Review of Economic Impacts and Provider Well-Being Outcomes
Source: Healthcare (Basel). 2025 Jul 7;13(13):1622. doi: 10.3390/healthcare13131622 (PMC12249292; doi:10.3390/healthcare13131622)
Supplement: Supplementary file 1 [file healthcare-13-01622-s001.zip › Supplementary table 1.docx]

**Supplementary Table S1:** Full search strategy per scientific database

| **PubMed** |
| --- |
| ((“Real?time”[tiab] AND “patient experience*”[tiab]) OR (“point?of?care”[tiab] AND “patient experience*”[tiab]) OR (“point?of?service”[tiab] AND “patient experience*”[tiab]) OR ((“online rating*”[tiab] OR “online comment*”[tiab] OR online feedback*[tiab]) AND “patient experience*”[tiab]) OR ("Social Media"[Major] AND “patient experience*”[tiab]) OR “Patient experience data”[tiab] OR “experience improvement*”[tiab] OR “improving experience*”[tiab] OR “experience feedback”[tiab] OR “guided tour*”[tiab] OR "experience round*"[tiab] OR "empathy map*"[tiab] OR "journey map*"[tiab] OR "design tool*"[tiab] OR "design technique*"[tiab] OR "always events*"[tiab] OR "narrative feedback"[tiab] OR "patient tracer*"[tiab] OR "Personal Narratives as Topic"[Major] OR "Narrative Medicine"[Major] OR "Medicine in the Arts"[Major] OR “patient narrative*”[tiab] OR “patient shadow*”[tiab] OR "patient stories"[tiab] OR photovoice[tiab] OR “Photo?elicitation”[tiab] OR “video diar*”[tiab] OR “patient diar*”[tiab] OR “process map*”[tiab] OR “suggestion box*”[tiab] OR “laddering interview*”[tiab] OR “hierarchical value map*”[tiab] OR “Consumer assessment of healthcare providers and systems”[tiab] OR “experience survey*”[tiab] OR ("patient complaints"[tiab] AND “patient experience”[tiab]) OR ("grievances"[tiab] AND “patient experience”[tiab]) OR “Patient?Reported?Experience*”[tiab] OR ((“free-text response*”[tiab] OR “free-text feedback*”[tiab] OR “free-text messages*”[tiab]) AND “patient experience*”[tiab]) OR "Natural Language Processing"[Major] OR "Data Mining"[Major] OR "Sentiment Analysis"[Major] OR "Machine Learning"[Major] OR "Data Visualization"[Major] OR "Data Management"[Major] OR "Hospital-Patient Relations"[Major] OR “experience-based”[tiab] OR “experience of care”[tiab] OR co-design*[ti] OR codesign*[ti]**) AND** ("Patient-Centered Care"[Mesh] OR “Patient Satisfaction"[Mesh] OR "Patient Reported Outcome Measures"[Mesh] OR “Patient Experience*”[tiab] OR “Patients Experience*”[tiab] OR “Family Experience*”[tiab] OR “Consumer Experience*”[tiab] OR “Consumers Experience*”[tiab] OR “Client Experience*”[tiab] OR “Clients Experience*”[tiab] OR "Health Communication"[Mesh] OR "Nonverbal Communication"[Mesh] OR "Professional-Family Relations"[Mesh] OR "Professional-Patient Relations"[Mesh] OR "Empathy"[Mesh] OR "Decision Making, Shared"[Mesh]) **AND** (Intervention*[tiab] OR tool*[tiab] OR "Quality Improvement"[Mesh] OR "Quality Assurance, Health Care"[Major] OR "Program Evaluation"[Major] OR "organization and administration"[Subheading] OR "Inservice Training"[Major] OR "Staff Development"[Major] OR “communication training*”[tiab] OR “communication skills training”[tiab] OR (“communication skills”[tiab] AND coaching[tiab]) OR "Hospital-Physician Relations"[Major] OR "Hospital-Patient Relations"[Major] OR "Hospital Communication Systems"[Major] OR “Mentoring”[Major] OR feedback[Major] OR "Clinical Audit"[Major] OR "Medical Audit"[Major] OR "education" [Subheading] OR improvement strategies[tiab] OR service improvement*[tiab] OR "Capacity Building"[Major] OR "Knowledge Management"[Major] OR "Data Science"[Major] OR "Medical Informatics"[Major] OR "Data Management"[Major] OR "Reminder Systems"[Major] OR "Information Dissemination"[Major] OR "Diffusion of Innovation"[Major] OR "Implementation Science"[Major] OR "Organizational Innovation"[Major] OR "Program Development"[Major] OR "Learning Health System"[Major]) **NOT** comment[pt] NOT protocol[ti] NOT "Editorial"[pt] **AND** ((systematic review[ti] OR systematic literature review[ti] OR systematic qualitative review[ti] OR systematic evidence review[ti] OR systematic quantitative review[ti] OR systematic meta-review[ti] OR systematic mixed studies review[ti] OR systematic mapping review[ti] OR systematic cochrane review[ti] OR systematic search and review[ti] OR “systematic review”[pt] OR (Cochrane Database Syst Rev[ta] AND review[pt]) OR ("Review" [pt] AND systematic[sb]) OR Realist review[ti] OR Realist synthesis[ti]) OR ("Clinical Study"[pt] OR "Feasibility Studies"[Mesh] OR "Pilot Projects"[Mesh] OR "Multicenter Study"[pt] OR "Control Groups"[Mesh] OR "Cross-Over Studies"[Mesh] OR "Comparative Study"[pt] OR "Evaluation Study"[pt])) **AND** English[language] |
| Filtered for |
| - Abstract |
| - Publications since Jan 1, 2015 |
|  |
| **CINAHL Plus with Full Text (through EBSCO)** |
| ((AB “Real?time” AND AB “patient experience*”) OR (AB “point?of?care” AND AB “patient experience*”) OR (AB “point?of?service” AND AB “patient experience*”) OR ((AB “online rating*” OR AB “online comment*” OR AB online feedback*) AND AB “patient experience*”) OR (MH "Social Media" AND AB “patient experience*”) OR AB “Patient experience data” OR AB “experience improvement*” OR AB “improving experience*” OR AB “experience feedback” OR AB “guided tour*” OR MM "Personal Narratives as Topic" OR MM "Narrative Medicine" OR MM "Medicine in the Arts" OR AB “patient narrative*” OR AB "patient stories" OR AB “patient shadow*” OR AB photovoice OR AB “laddering interview*” OR AB “hierarchical value map*” OR AB "experience round*" OR AB "empathy map*" OR AB "journey map*" OR AB "design tool*" OR AB "design technique*" OR AB "always events*" OR AB "narrative feedback" OR AB "patient tracer*" OR AB “Photo?elicitation” OR AB “video diar*” OR AB “patient diar*” OR AB “process map*” OR AB “suggestion box*” OR AB "grievances" OR AB “Consumer assessment of healthcare providers and systems” OR AB “experience survey*” OR (AB "patient complaints" AND AB “patient experience”) OR AB “Patient?Reported?Experience*” OR ((AB “free-text response*” OR AB “free-text feedback*” OR AB “free-text messages*”) AND AB “patient experience*”) OR MM "Natural Language Processing" OR MM "Data Mining" OR MM "Sentiment Analysis" OR MM "Machine Learning" OR MM "Data Visualization" OR MM "Data Management" OR MM "Hospital-Patient Relations" OR AB “experience-based” OR AB co-design OR AB codesign OR AB “experience of care”**) AND** (MH "Patient-Centered Care" OR MH “Patient Satisfaction" OR MH "Patient Reported Outcome Measures" OR AB “Patient Experience*” OR AB “Patients Experience*” OR AB “Family Experience*” OR AB “Consumer Experience*” OR AB “Consumers Experience*” OR AB “Client Experience*” OR MH "Health Communication" OR MH "Nonverbal Communication" OR MH "Professional-Family Relations" OR MH "Professional-Patient Relations" OR MH "Empathy" OR MH "Decision Making, Shared") **AND** (AB Intervention OR AB tool* OR MH "Quality Improvement" OR MM "Quality Assurance, Health Care" OR MM "Program Evaluation" OR MM "Inservice Training" OR MM "Staff Development" OR AB “communication training*” OR AB “communication skills training” OR (AB “communication skills” AND AB coaching) OR MM "Hospital-Physician Relations" OR MM "Hospital-Patient Relations" OR MM "Hospital Communication Systems" OR MM “Mentoring” OR MM feedback OR MM "Clinical Audit" OR MM "Medical Audit" OR AB improvement strategies OR AB service improvement* OR MM "Capacity Building" OR MM "Knowledge Management" OR MM "Data Science" OR MM "Medical Informatics" OR MM "Data Management" OR MM "Reminder Systems" OR MM "Information Dissemination" OR MM "Diffusion of Innovation" OR MM "Implementation Science" OR MM "Organizational Innovation" OR MM "Program Development" OR MM "Learning Health System")) **AND** ((TI systematic review OR TI systematic literature review OR TI systematic qualitative review OR TI systematic evidence review OR TI systematic quantitative review OR TI systematic meta-review OR TI systematic mixed studies review OR TI systematic mapping review OR TI systematic cochrane review OR TI systematic search and review OR PT “systematic review” OR (PT "Review" AND systematic[su]) OR TI Realist review OR TI Realist synthesis) OR (TI trial OR TI Study OR TI Evaluat* OR TI Compar* OR TI Feasibility OR TI Pilot OR TI Test* OR TI Control* OR TI Experiment* OR TI Quality Improvement OR SU Quality Improvement TI Program OR TI Intervention* OR TI Support* OR TI Systematic OR TI Approach OR TI Use OR TI Using OR TI Method* OR TI Research* OR TI Improv* TI Efficacy OR TI Effective*)) |
| Expanders |
| - Apply equivalent subjects |
| - Apply related words |
| Limiters |
| - Published Date: 20150101-20231231 |
| Source Types |
| - Academic Journals |
| Language |
| - English |
|  |
| **Scopus** |
| (TITLE-ABS-KEY ( "Real?time" W/3 experience ) OR TITLE-ABS-KEY ( "point?of?care" W/3 experience) OR TITLE-ABS-KEY ( "point?of?service" W/3 experience ) OR TITLE-ABS-KEY ( "online rating*" ) OR TITLE-ABS-KEY ( "online comment*" ) OR TITLE-ABS-KEY ( “online feedback*” ) OR TITLE-ABS-KEY ("patient" W/1 "experience") OR TITLE-ABS-KEY ( "patient" W/1 "experiences" ) OR TITLE-ABS-KEY ( "Social Media" W/6 "patient experience" ) OR TITLE-ABS-KEY ( "Patient experience data" ) OR TITLE-ABS-KEY ( "experience improvement*" ) OR TITLE-ABS-KEY ( "improving experience*" ) OR TITLE-ABS-KEY ( "experience feedback" ) OR TITLE-ABS-KEY ( "guided tour*" ) OR TITLE-ABS-KEY ( "experience round*" ) OR TITLE-ABS-KEY ("empathy map*" ) OR TITLE-ABS-KEY ( "journey map*" ) OR TITLE-ABS-KEY ( "design tool*" ) OR TITLE-ABS-KEY ( "design technique*" ) OR TITLE-ABS-KEY ( "always events*" ) OR TITLE-ABS-KEY ( "narrative feedback" ) OR TITLE-ABS-KEY ( "patient tracer*") OR TITLE-ABS-KEY ( "Patient Narrative*" ) OR TITLE-ABS-KEY ( "Patient Stories*" ) OR TITLE-ABS-KEY ( "Narrative Medicine" ) OR TITLE-ABS-KEY ( "patient shadow*" ) OR TITLE-ABS-KEY ( photovoice ) OR TITLE-ABS-KEY ( "laddering interview*" ) OR TITLE-ABS-KEY ( "hierarchical value map*" ) OR TITLE-ABS-KEY ( "CAHPS" ) OR TITLE-ABS-KEY ( "experience survey*" ) OR TITLE-ABS-KEY ( "patient complaints" W/3 experience ) OR TITLE-ABS-KEY ( "Patient Reported Experience*" ) OR TITLE-ABS-KEY ( “Photo elicitation” ) OR TITLE-ABS-KEY ( “video diar*” ) OR TITLE-ABS-KEY ( “patient diar*” ) OR TITLE-ABS-KEY ( “process map*” ) OR TITLE-ABS-KEY ( “suggestion box*” ) OR TITLE-ABS-KEY ( "free-text response*" W/9 "experience*") OR TITLE-ABS-KEY ( "free-text feedback*" W/4 "experience*" ) OR TITLE-ABS-KEY ( "free-text messages*" W/4 "experience*" ) OR TITLE-ABS-KEY ( "Natural Language Processing" ) OR TITLE-ABS-KEY ( "Data Mining" ) OR TITLE-ABS-KEY ( "Sentiment Analysis" ) OR TITLE-ABS-KEY ( "Machine Learning" ) OR TITLE-ABS-KEY ( "Data Visualization" ) OR TITLE-ABS-KEY ( "grievances" W/9 “patient experience”) OR TITLE-ABS-KEY ( "Data Management" ) OR TITLE-ABS-KEY ( "Hospital-Patient Relations" ) OR TITLE-ABS-KEY ( "experience-based" ) OR TITLE-ABS-KEY ( co-design ) OR TITLE-ABS-KEY ( codesign )) **AND** (TITLE-ABS-KEY ( "Patient-Centered Care" ) OR TITLE-ABS-KEY ("Patient-Centred Care" ) OR TITLE-ABS-KEY ( "Person-Centered Care" ) OR TITLE-ABS-KEY ( "Person-Centred Care" ) OR TITLE-ABS-KEY ( "Client-Centered Care" ) OR TITLE-ABS-KEY ( "Client-Centred Care" ) OR TITLE-ABS-KEY ( "Costumer-Centered Care" ) OR TITLE-ABS-KEY ( "Costumer-Centered Care" ) OR TITLE-ABS-KEY ( "User-Centered Care" ) OR TITLE-ABS-KEY ( "User-Centred Care" ) OR TITLE-ABS-KEY ( "Patient Satisfaction" ) OR TITLE-ABS-KEY ( "Patient Experience" ) OR TITLE-ABS-KEY ( "Patient Experiences" ) OR TITLE-ABS-KEY ( "Experience Measure*" ) OR TITLE-ABS-KEY ( "Experience survey*" ) OR TITLE-ABS-KEY ( "Family Experience*" ) OR TITLE-ABS-KEY ( "Consumer Experience*" ) OR TITLE-ABS-KEY ( "Consumers Experience*" ) OR TITLE-ABS-KEY ( "Client Experience*" ) OR TITLE-ABS-KEY ( "doctor* communication" ) OR TITLE-ABS-KEY ( "nurse* Communication" ) OR TITLE-ABS-KEY ( "provider communication" ) OR TITLE-ABS-KEY ( empathy ) OR TITLE-ABS-KEY ( "shared decision making" ) OR TITLE-ABS-KEY ( "communication skills" ) OR TITLE-ABS-KEY ( "experience data")) **AND** (TITLE-ABS-KEY ( Intervention) OR TITLE-ABS-KEY ( tool* ) OR TITLE-ABS-KEY ( "Quality Improvement" ) OR TITLE-ABS-KEY ( "Quality Assurance" ) OR TITLE-ABS-KEY ( "Program Evaluation" ) OR TITLE-ABS-KEY ( "Training" ) OR TITLE-ABS-KEY ( "Staff Development" ) OR TITLE-ABS-KEY ( coaching ) OR TITLE-ABS-KEY ( "Hospital-Patient Relation*" ) OR TITLE-ABS-KEY ( "Mentoring" ) OR TITLE-ABS-KEY ( "audit and feedback" ) OR TITLE-ABS-KEY ( "improvement cycle*" ) OR TITLE-ABS-KEY ( "improvement strategies" ) OR TITLE-ABS-KEY ( "service improvement*" ) OR TITLE-ABS-KEY ( "Capacity Building" ) OR TITLE-ABS-KEY ( "Knowledge Management" ) OR TITLE-ABS-KEY ( "Data Science" ) OR TITLE-ABS-KEY ( "Medical Informatics" ) OR TITLE-ABS-KEY ( "Data Management" ) OR TITLE-ABS-KEY ( "Reminder System*" ) OR TITLE-ABS-KEY ( "dashboard" ) OR TITLE-ABS-KEY ( "Information Dissemination" ) OR TITLE-ABS-KEY ( "Diffusion of Innovation" ) OR TITLE-ABS-KEY ( "Implementation Science" ) OR TITLE-ABS-KEY ( "Organizational Innovation" ) OR TITLE-ABS-KEY ( "Organizational development" ) OR TITLE-ABS-KEY ( "improvement journey" ) OR TITLE-ABS-KEY ( "Process Improvement" ) OR TITLE-ABS-KEY ( "Program Development" ) OR TITLE-ABS-KEY ( "Learning Health System")) **AND** ( LIMIT-TO ( PUBYEAR,2023) OR LIMIT-TO ( PUBYEAR,2021) OR LIMIT-TO ( PUBYEAR,2020) OR LIMIT-TO ( PUBYEAR,2019) OR LIMIT-TO ( PUBYEAR,2018) OR LIMIT-TO ( PUBYEAR,2017) OR LIMIT-TO ( PUBYEAR,2016) OR LIMIT-TO ( PUBYEAR,2015) ) **AND** ( LIMIT-TO ( DOCTYPE,"ar" ) ) AND ( LIMIT-TO ( LANGUAGE,"English" ) ) |
| Applied filters for sets of Keywords: |
| - ( LIMIT-TO ( EXACTKEYWORD,"Patient Satisfaction" ) OR LIMIT-TO ( EXACTKEYWORD,"Personal Experience" ) OR LIMIT-TO ( EXACTKEYWORD,"Patient Experience" ) OR LIMIT-TO ( EXACTKEYWORD,"Patient-Centered Care" ) OR LIMIT-TO ( EXACTKEYWORD,"Doctor Patient Relation" ) OR LIMIT-TO ( EXACTKEYWORD,"Physician-Patient Relations" ) OR LIMIT-TO ( EXACTKEYWORD,"Empathy" ) OR LIMIT-TO ( EXACTKEYWORD,"Doctor Patient Relationship" ) OR LIMIT-TO ( EXACTKEYWORD,"Experience" ) ) |
| - ( LIMIT-TO ( EXACTKEYWORD,"Interpersonal Communication" ) OR LIMIT-TO ( EXACTKEYWORD,"Patient-centered Care" ) OR LIMIT-TO ( EXACTKEYWORD,"Professional-Patient Relations" ) OR LIMIT-TO ( EXACTKEYWORD,"Professional-patient Relationship" ) ) |
| - ( LIMIT-TO ( EXACTKEYWORD,"Patient Satisfaction" ) OR LIMIT-TO ( EXACTKEYWORD,"Personal Experience" ) OR LIMIT-TO ( EXACTKEYWORD,"Patient Experience" ) OR LIMIT-TO ( EXACTKEYWORD,"Patient-Centered Care" ) OR LIMIT-TO ( EXACTKEYWORD,"Doctor Patient Relation" ) OR LIMIT-TO ( EXACTKEYWORD,"Physician-Patient Relations" ) OR LIMIT-TO ( EXACTKEYWORD,"Empathy" ) OR LIMIT-TO ( EXACTKEYWORD,"Doctor Patient Relationship" ) OR LIMIT-TO ( EXACTKEYWORD,"Experience" ) ) AND ( LIMIT-TO ( EXACTKEYWORD,"Interpersonal Communication" ) OR LIMIT-TO ( EXACTKEYWORD,"Patient-centered Care" ) OR LIMIT-TO ( EXACTKEYWORD,"Professional-Patient Relations" ) OR LIMIT-TO ( EXACTKEYWORD,"Professional-patient Relationship" ) ) AND ( LIMIT-TO ( EXACTKEYWORD,"Quality Improvement" ) OR LIMIT-TO ( EXACTKEYWORD,"Patient Participation" ) OR LIMIT-TO ( EXACTKEYWORD,"Communication Skill" ) OR LIMIT-TO ( EXACTKEYWORD,"Patient-reported Outcome" ) OR LIMIT-TO ( EXACTKEYWORD,"Patient Preference" ) OR LIMIT-TO ( EXACTKEYWORD,"Nurse Patient Relationship" ) OR LIMIT-TO ( EXACTKEYWORD,"Shared Decision Making" ) OR LIMIT-TO ( EXACTKEYWORD,"Patient Reported Outcome Measures" ) OR LIMIT-TO ( EXACTKEYWORD,"Satisfaction" ) OR LIMIT-TO ( EXACTKEYWORD,"Feedback System" ) OR LIMIT-TO ( EXACTKEYWORD,"Nurse-Patient Relations" ) ) |
| - ( EXCLUDE ( EXACTKEYWORD,"Medical Education" ) OR EXCLUDE ( EXACTKEYWORD,"Cross-sectional Study" ) ) |
|  |
| **PsycINFO (through EBSCO)** |
| ((AB “Real?time” AND AB “patient experience*”) OR (AB “point?of?care” AND AB “patient experience*”) OR (AB “point?of?service” AND AB “patient experience*”) OR ((AB “online rating*” OR AB “online comment*” OR AB online feedback*) AND AB “patient experience*”) OR (AB "Social Media" AND AB “patient experience*”) OR AB “Patient experience data” OR AB “experience improvement*” OR AB “improving experience*” OR AB “experience feedback” OR AB “guided tour*” OR AB "Patient stories" OR AB "Narrative Medicine" OR AB “patient narrative*” OR AB “patient shadow*” OR AB photovoice OR AB “laddering interview*” OR AB “hierarchical value map*” OR AB "experience round*" OR AB "empathy map*" OR AB "journey map*" OR AB "design tool*" OR AB "design technique*" OR AB "always events*" OR AB "narrative feedback" OR AB "patient tracer*" OR AB “Photo?elicitation” OR AB “video diar*” OR AB “patient diar*” OR AB “process map*” OR AB “suggestion box*” OR AB "grievances" OR AB “Consumer assessment of healthcare providers and systems” OR AB “experience survey*” OR (AB "patient complaints" AND AB “patient experience”) OR AB “Patient?Reported?Experience*” OR ((AB “free-text response*” OR AB “free-text feedback*” OR AB “free-text messages*”) AND AB “patient experience*”) OR AB "Natural Language Processing" OR AB "Data Mining" OR AB "Sentiment Analysis" OR AB "Machine Learning" OR AB "Data Visualization" OR AB "Data Management" OR AB "Hospital-Patient Relations" OR AB “experience-based” OR AB co-design OR AB codesign OR AB “experience of care”**) AND** (MH "Client Participation" OR MH “Client Satisfaction" OR MH "Patient Reported Outcome Measures" OR AB “Patient Experience*” OR AB “Patients Experience*” OR AB “Family Experience*” OR AB “Consumer Experience*” OR AB “Consumers Experience*” OR AB “Client Experience*” OR AB "Professional-Patient Communication" OR AB "Professional-Family Relations" OR AB "Professional-Patient Relations") **AND** (AB Intervention OR AB tool* OR MM “Quality of care” OR AB "Quality Improvement" OR AB "Quality Assurance" OR AB "Program Evaluation" OR AB "Inservice Training" OR AB "Staff Development" OR AB “communication training*” OR AB “communication skills training” OR (AB “communication skills” AND AB coaching) OR AB "Hospital Communication" OR AB “Mentoring” OR AB feedback OR AB "Clinical Audit" OR AB "Audit and Feedback" OR AB improvement strategies OR AB service improvement* OR AB "Capacity Building" OR AB "Knowledge Management" OR AB "Data Science" OR AB "Medical Informatics" OR AB "Data Management" OR AB "Reminder Systems" OR AB "Information Dissemination" OR AB "Diffusion of Innovation" OR AB "Implementation Science" OR AB "Organizational Innovation" OR AB "Program Development" OR AB "Learning Health System")) **AND** ((TI systematic review OR TI systematic literature review OR TI systematic qualitative review OR TI systematic evidence review OR TI systematic quantitative review OR TI systematic meta-review OR TI systematic mixed studies review OR TI systematic mapping review OR TI systematic cochrane review OR TI systematic search and review OR PT “systematic review” OR (PT "Review" AND systematic[su]) OR TI Realist review OR TI Realist synthesis) OR (TI trial OR TI Study OR TI Evaluat* OR TI Compar* OR TI Feasibility OR TI Pilot OR TI Test* OR TI Control* OR TI Experiment* OR TI Quality Improvement OR TI Program OR TI Intervention* OR TI Support* OR TI Systematic OR TI Approach OR TI Use OR TI Using OR TI Method* OR TI Research* OR TI Improv* TI Efficacy OR TI Effective*)) |
| Expanders |
| - Apply related words |
| - Apply equivalent subjects |
| Limiters |
| - Publication Year: 2015-2023 |
| - Peer-reviewed |
| Source Types |
| - Academic Journals |
| Language |
| - English |
|  |
| **Econlit (through EBSCO)** |
| (Patient* OR Health) AND ("Patient Experience*" OR "Inpatient Experience*" OR "Caregiver Experience*" OR "Caregivers experience*" OR "Consumer experience*" OR "Consumers experience*" OR "user experience*" OR "Users experience*" OR "patient feedback" OR "experience feedback" OR "experience survey*" OR "experience data") |
| Expanders |
| - Apply related words |
| - Apply equivalent subjects |
| Limiters |
| - Published Date: 20150101-20231231 |
| - Academic journals |
| **Subjects:** |
| - analysis of health care markets |
| - health behavior |
| - health: government policy; regulation; public health |
| - consumer economics: empirical analysis |
|  |
| **DOAJ (Directory of Open Access Journals)** – Search for articles Abstracts– filtered for Medicine and Social Sciences and for years of 2015 or later. |
| ("Patient-Centered Care" OR "Patient-Centred Care" OR "Person-Centered Care" OR "Person-Centred Care" OR "Client-Centered Care" OR "Client-Centred Care" OR "Costumer-Centered Care" OR "Costumer-Centered Care" OR "User-Centered Care" OR "User-Centred Care" OR "Patient Satisfaction" OR "Patient Experience*" OR "Experience Measure*" OR "Experience survey*" OR "Family Experience*" OR "Consumer Experience*" OR "Consumers Experience*" OR "Client Experience*" OR "doctor* communication" OR "nurse* Communication" OR "provider communication" OR empathy OR "shared decision making" OR "communication skills" OR "experience data") AND ( Intervention OR tool* OR "Quality Improvement" OR "Quality Assurance" OR "Program Evaluation" OR "Training" OR "Staff Development" OR coaching OR "Hospital-Patient Relation*" OR "Hospital Communication" OR "Mentoring" OR "audit and feedback" OR "improvement cycle*" OR "improvement strategies" OR "service improvement*" OR "Capacity Building" OR "Knowledge Management" OR "Data Science" OR "Medical Informatics" OR "Data Management" OR "Reminder System*" OR "dashboard" OR "Information Dissemination" OR "Diffusion of Innovation" OR "Implementation Science" OR "Organizational Innovation" OR "Organizational development" OR "improvement journey" OR “Process Improvement” OR "Program Development" OR "Learning Health System") |
