# Supplementary material for: The Value of Interventions Aimed at Improving the Patient Experience: Systematic Review of Economic Impacts and Provider Well-Being Outcomes
Source: Healthcare (Basel). 2025 Jul 7;13(13):1622. doi: 10.3390/healthcare13131622 (PMC12249292; doi:10.3390/healthcare13131622)
Supplement: Supplementary file 1 [file healthcare-13-01622-s001.zip › Supplementary Table 2_V3.docx]

**Supplementary Table S2:** Study design and methodological weaknesses based on the respective quality assessment checklists for the studies responding to the first study question.

|  | Study Design & Context | Overall Study Design – weaknesses / risk of bias (synthesis) | Economic Evaluation Component: risk of bias (synthesis) |
| --- | --- | --- | --- |
| (Schreiter et al., 2021) | Historical cohort of matched controls at patient level. Academic hospital, transition from surgery; University of Wisconsin Hospital, Wisconsin, USA. | No randomization. Historical controls, even though matched for some clinical indicators. Single site. No baseline outcomes assessed. Unadjusted analyses for patient experience outcome. No correction for multiple comparisons. | Margins calculated based on estimated (versus actual) reimbursement. The lack of an incremental cost-effectiveness ratio is offset by the non-significant between-group differences on the operation margin, and the incremental cost per patient is reported. |
| (Abu-Ghname et al., 2021) | Pre-post test, retrospective, one site. Surgeons (*n*= 56); controlled subgroup analysis for plastic surgeons (*n*= 8); pediatric emergency department, Children’s Hospital, Texas, USA. | No control group. Retrospective assessment. Single organization. No covariate adjustment. No individual-level analysis. Small sample for the controlled subgroup analysis. | Costs of communication course and billable services were not considered; only revenue (charges and payments) was calculated. No caution reported regarding generalizability. |
| (Sharma et al., 2020) | Longitudinal (2007–2014), retrospective comparative study (secondary analysis). 132 US acute care hospitals. Key-informant interviews with staff (*n*> 13) of a teaching hospital. | Retrospective study. No details (e.g., employees, budgets) of the patient-experience office structures, but years of operation of an office were accounted for. Study period covers early adopters, and the follow-up was not sufficient for determining curvilinear effects. | No incremental analysis of costs and consequences related to effectiveness. Only total operating costs examined and not direct costs of adding and operating a patient experience office. No sensitivity analysis for cost models. |
| (Thum et al., 2022) | Pre- and post-test, one organization with multiple units; academic tertiary care center and its affiliated community hospital; Thomas Jefferson University Hospital and affiliate, USA. | No control group. Eligibility criteria not clearly described. Single pretest (although long preintervention period). No establishment of stable baseline through multiple measurement time points. No report on the numbers of surveys used for the analysis, only those mailed. No correction for multiple comparisons. | Unspecified / unreported methods for the revenue impact analysis. |
