# Supplementary material for: The Value of Interventions Aimed at Improving the Patient Experience: Systematic Review of Economic Impacts and Provider Well-Being Outcomes
Source: Healthcare (Basel). 2025 Jul 7;13(13):1622. doi: 10.3390/healthcare13131622 (PMC12249292; doi:10.3390/healthcare13131622)
Supplement: Supplementary file 1 [file healthcare-13-01622-s001.zip › Supplementary Table 3_V3.docx]

**Supplementary Table S3:** Study design and respective methodological weaknesses based on quality assessment checklists for the studies responding to the second review question.

|  | Study Design | Study Design – methodological weaknesses / risk of bias (synthesis) |
| --- | --- | --- |
| (LaBedz et al., 2022) | Pragmatic RCT (patient-level randomization). Multivariable linear regression models, with a Bonferroni correction for the co-primary outcomes, sensitivity analyses for missing data imputation, and exploratory analyses for heterogeneity of treatment effects. Minority-serving hospital with a transition to home; University of Illinois Hospital & Health Sciences System, Chicago, Illinois, USA. | Single site. Patients not masked to group allocation. Limited fidelity of the implementation: only 29% of navigator group participants received the intervention per protocol. While the authors use a standardized measure, it is not a traditional patient experience measure (i.e., focus on social support dimensions partly addressed by the intervention) and may not be specific or responsive enough for the intervention. |
| (Altamirano et al., 2022) | Pre-post test (3 months post-intervention), four sites. Physicians (*n*= 104; 22% response rate). | No control group, but 4 sites enabling subgroup analysis. Self-selected trainees, but used propensity scores to partly mitigate this risk. No Bonferroni correction for multiple items assessed, affecting the significancy of the results. |
| (March et al., 2022) | Pre- and post- test, single-center retrospective review of a pilot program. χ2 test on the top-box score after T test on baseline characteristics. Hospital, pharmacy; Methodist University Hospital, Memphis, Tennessee, USA | Retrospective, single-site pre-post-test analysis not adjusted for covariates. No establishment of stable baseline through multiple measurement time points. No correction for multiple comparisons. Intervention not delivered to all eligible patients (e.g., not during weekends). No report on the number of patient experience surveys collected. Risks of selected findings: two different designs for two outcomes. |
| (Congiusta et al., 2020) | Pre-post test (burnout outcomes) within a RCT (patient experience outcomes), trained physicians (*n*= 30). | No control groups for the burnout outcomes. Overall study (on the patient experience outcomes) with no baseline outcomes differences tested, no data on covariates, small sample sizes leading to non-parametric statistical testing, and self-selected participants. |
| (Boissy et al., 2016) | Pre-post design, including 3-month follow-up – burnout outcomes (controlled, before- and-after for the patient experience outcomes). Physicians (*n*= 947; 147 with 3-month follow-up data) | No controls for the burnout outcome. Overall study (on patient experience outcomes) with no randomization, heterogenous controls, no difference-in-differences analysis, and possible contamination. |

Note: (Schreiter et al., 2021) (Thum et al., 2022) cumulatively fit the first and second review question. The methods and appraised limitations are described in Supplementary Appendix 2.
